# Microbial Community Composition and Functional Capacity in a Terrestrial Ferruginous, Sulfate-Depleted Mud Volcano

**DOI:** 10.3389/fmicb.2017.02137

**Published:** 2017-11-02

**Authors:** Tzu-Hsuan Tu, Li-Wei Wu, Yu-Shih Lin, Hiroyuki Imachi, Li-Hung Lin, Pei-Ling Wang

**Affiliations:** ^1^Institute of Oceanography, National Taiwan University, Taipei, Taiwan; ^2^Department of Geosciences, National Taiwan University, Taipei, Taiwan; ^3^Department of Subsurface Geobiological Analysis and Research, Japan Agency for Marine-Earth Science and Technology, Yokosuka, Japan; ^4^The Experimental Forest, College of Bio-Resources and Agriculture, National Taiwan University, Nantou, Taiwan; ^5^Department of Oceanography, National Sun Yat-sen University, Kaohsiung, Taiwan; ^6^Research and Development Center for Marine Resources, Japan Agency for Marine-Earth Science and Technology, Yokosuka, Japan

**Keywords:** mud volcano, methane, AOM, methanogenesis, ANME, Taiwan

## Abstract

Terrestrial mud volcanoes (MVs) are an important natural source of methane emission. The role of microbial processes in methane cycling and organic transformation in such environments remains largely unexplored. In this study, we aim to uncover functional potentials and community assemblages across geochemical transitions in a ferruginous, sulfate-depleted MV of eastern Taiwan. Geochemical profiles combined with 16S rRNA gene abundances indicated that anaerobic oxidation of methane (AOM) mediated by ANME-2a group coincided with iron/manganese reduction by Desulfuromonadales at shallow depths deprived of sulfate. The activity of AOM was stimulated either by methane alone or by methane and a range of electron acceptors, such as sulfate, ferrihydrite, and artificial humic acid. Metagenomic analyses revealed that functional genes for AOM and metal reduction were more abundant at shallow intervals. In particular, genes encoding pili expression and electron transport through multi-heme cytochromes were prevalent, suggesting potential intercellular interactions for electron transport involved in AOM. For comparison, genes responsible for methanogenesis and degradation of chitin and plant-derived molecules were more abundant at depth. The gene distribution combined with the enhanced proportions of 16S rRNA genes related to methanogens and heterotrophs, and geochemical characteristics suggest that particulate organic matter was degraded into various organic entities that could further fuel *in situ* methanogenesis. Finally, genes responsible for aerobic methane oxidation were more abundant in the bubbling pool and near-surface sediments. These methane oxidizers account for the ultimate attenuation of methane discharge into the atmosphere. Overall, our results demonstrated that various community members were compartmentalized into stratified niches along geochemical gradients. These community members form a metabolic network that cascades the carbon transformation from the upstream degradation of recalcitrant organic carbon with fermentative production of labile organic entities and methane to downstream methane oxidation and metal reduction near the surface. Such a metabolic architecture enables effective methane removal under ferruginous, sulfate-depleted conditions in terrestrial MVs.

## Introduction

Mud volcanoes (MVs) are prominent surface expressions in compressional tectonic regimes (Kopf, 2002). They are morphologically diverse, ranging from mud pies to conical structures, from which gaseous fluids with unconsolidated, fine-grained sediments are expelled (Dimitrov, 2002; Kopf, 2002). Often, methane with CO_2_ and minor amounts of C_2+_ hydrocarbons constitute the emitted phase (Mazzini et al., 2009). Recent estimates have revealed that MVs and seepages onshore and offshore might account for 27–36% of methane emissions from natural sources (Reeburgh, 2007; Etiope et al., 2008). Considering the potentially huge storage of methane in subsurface environments (Milkov et al., 2003), uncovering the regulative mechanisms involved in methane cycling and related metabolisms would enable better assessments of methane emission over various time scales (Mazzini and Etiope, 2017).

Net methane emission in terrestrial MVs is governed by physical and chemical characteristics of fluid reservoir, transportation styles, fluid pathways, and *in situ* microbial activities (Mazzini and Etiope, 2017). Unlike their marine counterparts, whose emissions are mitigated by aerobic methane oxidation in seawater above them, terrestrial MVs emit methane directly into the atmosphere. Previous studies have indicated that the exact quantity of methane emission is primarily controlled by *in situ* microbial production and consumption near the surface (Alain et al., 2006; Chang et al., 2012; Cheng et al., 2012; Wrede et al., 2012; Wang P.-L. et al., 2014). The general framework for such microbial control has been speculated to share similarity with soil ecosystems where the decomposition of detrital organic carbon or dead plant biomass produces CO_2_ or small organic compounds that could be converted into methane under strictly anoxic conditions (Achtnich et al., 1995; Davidson and Janssens, 2006). Methane is either directly released into the atmosphere or oxidized with electron acceptors within sediments or from surface (e.g., oxygen, sulfate, or metal oxides) (Cheng et al., 2012; Wang P.-L. et al., 2014). Although the fate of methane produced *in situ* has been demonstrated in previous studies (Cheng et al., 2012; Wang P.-L. et al., 2014), the assemblage and distribution of community members responsible for upstream organic mineralization and production of precursors (e.g., H_2_, acetate, and methyl-compounds) for methanogenesis in terrestrial MV environments have not been fully revealed.

Most terrestrial MVs are characterized by a limited availability of sulfate (<1 mM). Such a low quantity of sulfate would increase the free energy (less negative) and drive sulfate-dependent anaerobic oxidation of methane (AOM) less energetically favorable. Therefore, whether the syntrophic partnerships between ANME groups and sulfate reducing *Desulfosarcina/Desulfococcus* or *Desulfobulbus* clusters commonly observed in sulfate-rich marine settings (Knittel and Boetius, 2009) could be maintained or succeeded with different cell organization and metabolic pathway is not clear. On the basis of geochemical characteristics, incubation experiments, and microscopic observations, AOM using electron acceptors other than sulfate, such as ferric oxides, manganese oxides, nitrate, and humic acids, have been proposed or validated for marine sediments, freshwater sediments, wetland sediments, or anaerobic wastewater sludge (Beal et al., 2009; Haroon et al., 2013; Deutzmann et al., 2014; Egger et al., 2014; Ettwig et al., 2016; Scheller et al., 2016; Valenzuela et al., 2017). The feasibility of these electron acceptors for AOM catalyzed by a given community has not been demonstrated for terrestrial MV environments.

The aim of this study is to uncover the gene and community compositions involved in methanogenesis and methane oxidation, and the effect of various electron acceptors on AOM activity in a terrestrial ferruginous, sulfate-depleted MV. The Lei-Gong-Hou (LGH) MVs in eastern Taiwan were chosen for this investigation because previous studies have indicated that AOM mediated by the ANME-2a group co-occurred with iron/manganese reduction potentially catalyzed by *Desulfuromonas* spp. and *Pelobacter* spp. under low-sulfate conditions (Chang et al., 2012; Wang P.-L. et al., 2014). The switchable interlayering of AOM and methanogenesis with respect to depth further highlights that *in situ* methanogenesis driven by the decomposition of carbohydrate is essential to supply methane for AOM at adjacent depths. To further investigate the potential of AOM, and microbial community composition and functional capacity across geochemical gradients, we collected geochemical and molecular data for a sediment core, and performed incubations of mud slurries supplied with ^13^C-labeled methane and a variety of terminal electron acceptors. These data sets were further combined and integrated to demonstrate the flexibility of AOM processes, and the gene patterns and community compositions involved in methane and organic cycling.

## Materials and Methods

### Sample Collection and Processing

Muddy fluids from a bubbling pool (named LGH03) and a core with a total length of 160 cm from the pool margin were retrieved from the LGHMVs in eastern Taiwan in July of 2011 for geochemical and molecular analyses. Fluids from another bubbling pool (named LGH09) and sediments at a depth of 10 cm on the pool margin were collected and mixed at a ratio of 1:1 in a serum bottle with no headspace left in August of 2015 for incubations. Geological and site backgrounds of LGHMVs have been described in previous studies (Chang et al., 2012; Wang P.-L. et al., 2014). After retrieval, samples were transported to the laboratory within 5 h and the core was immediately sectioned at 5-cm intervals for analyses of gas and aqueous geochemistry, and DNA. The represented depth was the average depth of individual sectioned intervals. For gas geochemistry, a total of 6 mL of sediments from sectioned sediments was preserved in a serum bottle filled with 10 mL of 1 M NaOH, and sealed with a butyl rubber stopper and an aluminum ring. The remaining sediments were subject to centrifugation at 8,200 × *g* for 15 min for aqueous geochemistry. Supernatants were filtered through 0.22-μm pore-sized polyethersulfone filters and split into four fractions. The cation fraction was preserved with 1/10 by volume of 2 M nitric acid. The fractions for anions and dissolved organic carbon (DOC) individually were stored in pre-washed vials without any preservative at 4°C (for anions) or -20°C (for DOC). The fraction for dissolved inorganic carbon (DIC) was sealed in serum bottles and acidified with phosphorous acid.

### Geochemical Analysis

Concentrations of dissolved Fe and Mn were measured using an Ultima2 inductively coupled plasma-optic emission spectrometer (HORIBA Jobin Yvon, United States). Concentrations of anions were determined using an ion chromatograph (ICS-3000, Dionex, United States). DOC was oxidized to CO_2_ through thermal catalytic conversion and measured using a Shimadzu TOC analyzer (TOC-L). The abundances of DIC and gaseous hydrocarbon compounds in headspace were measured using a gas chromatograph (GC) equipped with a Porapak Q column, a thermal conductivity detector, and a flame ionization detector (6890N, Agilent Taiwan, Taiwan). The measured partial pressure of a specific gas compound was converted into the dissolved concentration with the volume of pore water. Concentrations of particulate total organic carbon (TOC) were determined by an elemental analyzer (EA, vario MICRO cube, Elementar). Carbon isotopic compositions of DIC, methane, and TOC were determined using a GC IsoLink or Flash EA in line with a MAT 253 isotope ratio mass spectrometer (Thermo Fisher Scientific, United States). The isotopic compositions were reported using the δ notation (in permil, ‰):

$$\delta^{13}C=[{{(}^{13}{C/}^{12}C)}_{\mathrm{sample}}/{{(}^{13}{C/}^{12}C)}_{\mathrm{standard}}-1]\;\times \;1000$$

where the standard is referred to Vienna Pee Dee Belemnite (VPDB). The uncertainties for aqueous and gas geochemistry, elemental abundance of TOC, and δ^13^C are ±2%, ±5%, ±2%, and ±0.3‰, respectively. The detectable limits for anions and cations with the consideration of dilution were 10 ppm and 0.1 ppm, respectively. Spearman’s correlation was employed to examine the relationships between environmental factors.

### DNA Extraction

Crude DNA for 16S rRNA gene and metagenomic analyses of environmental samples was extracted from 10 g of fluids/sediments in the bubbling pool (0 cm) and eleven depth intervals (2.5, 7.5, 12.5, 17.5, 27.5, 47.5, 67.5, 87.5, 107.5, 127.5, and 157.5 cm) of the core using the Ultraclean Mega DNA Prep Soil Kit (MoBio, United States). Crude DNA for 16S rRNA gene analyses of incubated samples was extracted from one gram of incubated slurry using the PowerSoil DNA Isolation Kit (MoBio, United States). After extraction, genomic DNA was purified and concentrated by a OneStep PCR Inhibitor Removal Kit and a Genomic DNA Clean and Concentrator Kit (Zymo Research, United States) and stored at -80°C for subsequent analyses.

### 16S rRNA Gene Amplicon Sequencing

High-throughput sequencing of dual-indexed PCR amplicons encompassing the V4 region of 16S rRNA gene was used to assess community compositions along depth and in incubated slurries. Fragments of 16S rRNA genes were amplified using the primer combinations of F515 (5′–GTG CCA GCM GCC GCG GTA A–3′) and R806 (5′–CCC GTC AAT TCM TTT RAG T–3′) that target both bacterial and archaeal communities (Kozich et al., 2013). Both forward and reverse primers were barcoded and appended with the Illumina-specific adapters. Each PCR mixture contained 1.1–1.5 ng of purified genomic DNA, 1 U of ExTaq polymerase (TaKaRa Bio, Japan), 0.2 mM of dNTPs, 0.2 μM of each primer, and 5 μl of 10× PCR buffer in a total volume of 25 μL. Thermal cycling involved a denaturation step at 94°C for 3 min followed by 30 cycles of denaturation at 94°C for 45 s, annealing at 55°C for 45 s, extension at 72°C for 90 s, and a final extension step at 72°C for 10 min. The products of three independent PCRs for individual samples were pooled, analyzed by gel electrophoresis for size verification (∼ 400 bp), and purified using the DNA Clean and Concentrator Kit (Zymo Research, United States). Amplicons from different samples were pooled in equal quantities sufficient for sequencing on an Illumina MiSeq platform (Illumina, United States).

### 16S rRNA Gene Copy Number

Quantitative PCR was used to analyze the 16S rRNA gene copy number of bacteria, archaea, and ANME-2a in the environmental samples and incubated mud slurries using a MyiQ Real-time PCR Detection System (Bio-Rad, United States). All samples were analyzed in triplicate reactions (20 μl each) with each composed of 1× SsoFast EvaGreen Supermix (Bio-Rad, United States), 100 nM of each primer, and 2 μL of template DNA. Primers and PCR conditions were the same as those described in Chang et al. (2012) and Wang P.-L. et al. (2014). The 16S rRNA gene copy number of a specific group was calculated assuming 650 g mole^-1^ of one base pair of DNA.

### Sequence Analysis of 16S rRNA Gene Amplicons

Sequences of 16S rRNA gene amplicons were analyzed using the Mothur 1.34 following the standard protocols (Schloss et al., 2009). Barcoded sequences were de-multiplexed and filtered to remove low quality reads (Phread score < 25). Reads that had more than two mismatches to the paired barcode sequences were removed by the noise reduction while retaining the information of the representative reads and the numbers of reads merged by pre-clustering (Huse et al., 2010). The unique reads were aligned to the Silva NR119 database^1^. Reads not aligned in the same region were removed. The sequence regions beyond the primers were truncated. Potential chimeric sequences were detected and removed using the UCHIME program (Edgar et al., 2011). The number of sequences in each sample after quality filtering is shown in Supplementary Table S1.

The taxonomy of each unique sequence was assigned using the Silva SSU dataset of the NR 119 release as the reference. Taxonomy assignments with bootstrap values greater than 80% were considered to be valid. Sequences sharing more than 97% identity were further clustered into individual operational taxonomy units (OTUs) using the nearest neighbor algorithm (Schloss and Westcott, 2011). Based on the rarefied datasets (*n* = 10,008), alpha diversity indices, such as the number of observed OTUs, Chao-1, and Inverse Simpson (Hill, 1973; Chao et al., 1984; Faith, 1992), were computed.

The weighted Unifrac (Lozupone and Knight, 2007), which quantitatively incorporates the relatedness of community members with their abundances for the evaluation of difference between samples, was used to determine the community dissimilarity between samples from the sediment core. In addition, analyses of non-metric multidimensional scaling (NMDS) was conducted on the basis of the weighted Unifrac distance matrix using the software Mothur 1.34 (Schloss et al., 2009). The significance of environmental variables relative to the NMDS ordinations was computed using “envfit” and 999 permutations. Finally, the dissimilarity matrix between samples from the sediment core and incubations was constructed by the Bray–Curtis method (Bray and Curtis, 1957) and visualized by principal coordinate analysis (PCoA) using the R “Phyloseq” package.

### Analysis for Metagenomes

Based on the geochemical characteristics of pore fluids and gases, crude DNA extracted from samples from the bubbling pool and 2.5, 17.5, 27.5, 47.5, 87.5, and 127.5 cm of the core was selected for metagenomic analyses. In brief, metagenomic libraries with an insert size of 200–600 bp and 50 ng of DNA of each sample were prepared using the Ovation Ultralow System V2 1-96 (Nugen Technologies, United States). Paired-end sequencing (2× 300 bp) was performed on an Illumina Miseq platform.

Low quality reads with a Phread score below 25, a length longer or shorter than two standard deviations from the mean, and ambiguous bases (Ns) were filtered from the dataset by the Mothur (Schloss et al., 2009; Hurwitz et al., 2013). Reads 1 and 2 from the same fragment were subsequently merged. Since duplicate reads sharing 100% nucleotide similarity and identical length may represent sequencing artifact, the filtered reads were treated by digital normalization to systematize the data coverage, thereby decreasing the sampling variation, discarding redundant data, and removing the majority of errors (Brown et al., 2012).

Metagenomic sequences were compared using the DIAMOND BLASTX (Buchfink et al., 2015) against the NCBI-nr database of non-redundant protein sequences (as of March 2015). BLASTX matches to prokaryote genes (Bacteria and Archaea) above a bit score of 60 and a minimum length of 50 bp were retained and evaluated according to functional categories based on the SEED classification of functional roles and subsystems (Overbeek et al., 2005). The taxonomic composition of protein-coding genes was determined using the MEtaGenome ANalyzer 5 (Huson et al., 2011) based on the annotations of BLASTX-identified genes in accordance with the NCBI taxonomy.

To further evaluate genes involved in methane metabolism, degradation of lignocellulose and chitin, fermentation, hydrogen production and consumption, dissimilatory metal reduction, dissimilatory sulfate reduction, sulfur oxidation, dissimilatory nitrogen reduction, and nitrogen fixation, a list of key enzymes/functional genes (Supplementary Table S2) was searched against the results of BLASTX matching coding genes in the NCBI-nr database (the bit score is above 60 and the minimum length is 50 bp) by custom scripting. Since genes responsible for metal reduction have not been well determined, four genes including cytochrome c peroxidase (*mac*A), sigma-54-dependent transcriptional response regulator (*pil*R), cytochrome C biogenesis protein (*ppc*A), and doubled CXXCH motif (*CxxCH*) were targeted to investigate the potential capability of metal reduction (Beliaev and Saffarini, 1998; Butler et al., 2004; Shi et al., 2007; Liu et al., 2012; Kim et al., 2014). A customized database composed of available complete genomes of methanogens and partial genomes of ANME groups was used for the annotation and determination of their taxonomy and relative abundances of genes related to methanogenesis and AOM. Genes used for these analyses are the key genes (including *mcr, mtr, fwd, mch, mer*, and *mtd*) for methanogenesis (Thauer, 2011). Additionally, multi-heme cytochrome (MHC) genes with predicted S-layer domains from reconstructed ANME-2a genomes (ANME-2a: 2566125052 and 2566123487) (McGlynn et al., 2015) were tagged to represent the potential for direct interspecies electron transfer (DIET).

Gene-centric analysis was used to reveal differences in community functional capacity (Gill et al., 2006). First, 37 single-copy housekeeping genes (Supplementary Table S3) were used to evaluate whether any metagenome was over- or under-represented by the sequencing bias (He et al., 2015). To do this, the odds ratios were calculated by dividing the abundances of specific single-copy genes in individual metagenomes with those in a combined average metagenome. For odds ratios around one, the sequencing bias for obtained metagenomes was minimal.

The odds ratios of the abundances of individual functional genes in individual metagenomes to that in the combined average metagenome were used to compare gene abundances across samples and displayed with a heat map using the R “gplots” package. The average gene length based on the full-length opening reading frames from sequenced genomes deposited on NCBI (Supplementary Table S2) and the total number of reads matching coding genes in the NCBI-nr database in each sample (Supplementary Table S1) were first used to normalize the number of each functional gene (Supplementary Table S2). Then, the number of sequences matching the universal, putative single-copy gene encoding RNA polymerase subunit B (*rpo*B, 4020 bp) was used to normalize the sequence count of target genes. A value equal to one indicates that the abundance of specific genes in the metagenome is equivalent to that of *rpo*B.

All sequence data generated in this study are publicly available in the NCBI database under the BioProject accession PRJNA386622.

### Incubation Experiments for AOM

A small portion of collected mud slurries was drained and the headspace was replaced with 100% methane at ∼1.5 bar. The bottle was kept at room temperature in an anoxic chamber (Coy Laboratory Products, United States) for pre-incubation for 3 days to reduce any possible oxygen interference and stimulate the activity of anaerobic methane oxidizer. The basal salt solution was prepared in accordance with the general geochemical characteristics of the site following the recipe reported in Cheng et al. (2014) with a chloride concentration adjusted to 500 mM. The reducing agent (Na_2_S × 9H_2_O) at a final concentration of 2.08 mM was added into the sterilized basal salt solution to decrease the redox potential and remove any trace amount of oxygen. The basal salt medium was sulfate free and did not contain any carbon source or electron acceptor.

For every set of incubations, slurries were mixed with an equal volume of basal salt and capped with butyl rubber stoppers and aluminum rings. Triplicate bottles were supplemented with sulfate (10 mM), nitrate (5 mM), anthraquinone-2,6-disulfonate (AQDS, 1 mM), ferrihydrite (10 mM), fumarate (1 mM), ferrihydrite with AQDS (10 and 1 mM), ferrihydrite with fumarate (10 and 1 mM), or no exogenous electron acceptors (Supplementary Table S4). Ferrihydrite was synthesized by neutralizing a 0.4 M solution of ferric chloride (FeCl_3_) with sodium hydroxide (NaOH) (Lovley and Phillips, 1986). The headspace gas was first exchanged in three cycles of vacuum and 0.22-μm filtered nitrogen with an end pressure of 1.01 bar. Then, 2 mL of 99.99% ^13^CH_4_ (Campro Scientific, Veenendaal, Netherlands) was injected into the headspace of each vial to provide a concentration of ∼1.82 × 10^4^ ppmv. Additionally, two sets of controls were prepared. One was a nitrogen control (slurries mixed with nitrogen at 1.01 bar in the headspace), whereas the other was a sterilized control prepared with autoclave-sterilized slurries and ^13^CH_4_ in duplicate. In total, eight sets of incubations and two controls were performed under dark conditions at room temperature. The pH of resultant mixture was measured using the pH test strip and ranged from 7 to 8.

The total amounts of ^13^CO_2_ in headspace and its isotopic compositions were monitored at a 30-55 day interval using a gas chromatography in line with a MAT 253 isotope ratio mass spectrometer. The headspace concentration was used to calculate the equilibrium concentration of DIC using the Henry’s law constant (Stumm and Morgan, 1996). The total moles of ^13^CO_2_ in headspace and dissolved form were summed and plotted against time duration of incubation. Methane oxidation rates were calculated by applying a linear regression on the accumulated amount of ^13^CO_2_ versus the time interval. The calculation assumed that all the produced ^13^CO_2_ in headspace was in equilibrium with the dissolved form and was solely derived from AOM. To compare whether the AOM rate of a specific treatment was significantly different from that for the sterilized control, analysis of covariance (ANCOVA) was conducted.

## Results

### Geochemical Characteristics

**FIGURE 1:**
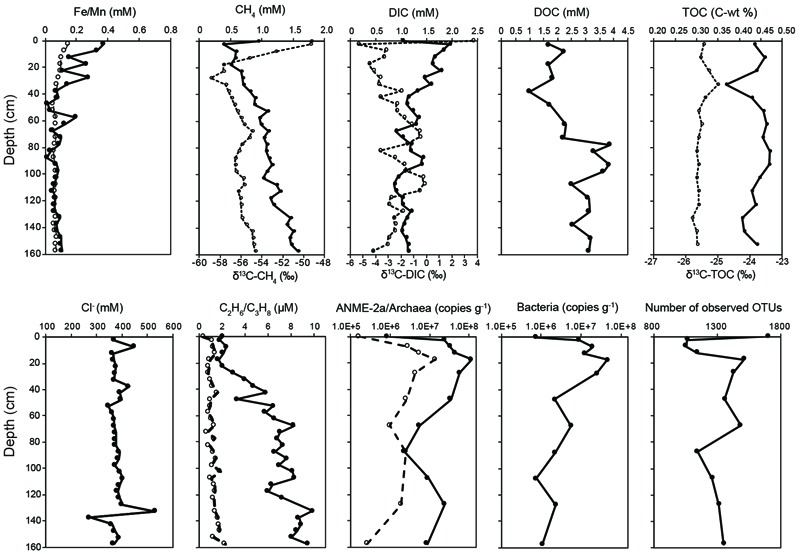
Porewater geochemistry, 16S rRNA gene abundance, and community diversity revealed by 16S rRNA gene amplicon. The concentrations of dissolved Fe, methane, DIC, TOC, and ethane are labeled with solid circles, whereas the δ^13^C values of CH_4_, DIC, and TOC, and concentrations of dissolved Mn and propane are labeled with open circles. Target taxonomic units for qPCR analyses include archaea (solid circles), ANME-2a (open circles), and bacteria (solid circles). The unit of qPCR analyses is copies per gram of wet sediments. Data points at 0 cm represent the characteristics of the bubbling fluids.

Geochemical profiles of pore water showed various characteristics related to abiotic and microbial processes (**Figure 1** and Supplementary Table S5). Chloride concentrations ranged between 274 and 532 mM, with most data clustering between 350 and 400 mM and great fluctuations occurring at 7.5, 37.5, and 130–140 cm depth. Dissolved manganese and iron concentrations decreased with depth, with low values observed generally below 60 cm. Concentrations of sulfate and other major anions were below the detectable level. Methane concentrations increased from 0.39 to 1.57 mM with increasing depth. Carbon isotopic compositions of methane decreased from -49‰ at the core top to -59‰ at 25 cm and increased gradually to -55‰ at the core bottom. DOC concentrations were low (between 1 and 2.3 mM) at <72.5 cm and increased to above 2.5 mM at >72.5 cm. For comparison, DIC concentrations decreased from 1.9 to 1.1 mM with depth. The δ^13^C values of DIC generally increased from -5.4‰ at the core top to -0.2‰ at 107.5 cm and decreased to -4.3‰ at the core bottom.

### Abundances of 16S rRNA Genes

Abundances of all investigated taxonomic groups varied by two to three orders of magnitude along depth (**Figure 1**). The bacterial 16S rRNA gene abundances ranged between 7.33 × 10^5^ and 4.61 × 10^7^ copies (g^-1^ of sediments). Along depth, the copies of bacterial 16S rRNA genes were the lowest in the bubbling pool, increased to a maximum of 4.61 × 10^7^ copies (g^-1^ of sediments) at 17.5 cm, and then exhibited a decline of one to two orders of magnitude toward the core bottom. The abundances of archaeal 16S rRNA genes ranged between 8.39 × 10^5^ and 1.19 × 10^8^ copies (g^-1^ of sediments), whereas the abundances of ANME-2a 16S rRNA genes varied from 1.67 × 10^5^ to 1.41 × 10^7^ copies (g^-1^ of sediments). Their variations were generally comparable with those of bacteria.

### Environmental Controls on Community Diversity and Structure

**FIGURE 2:**
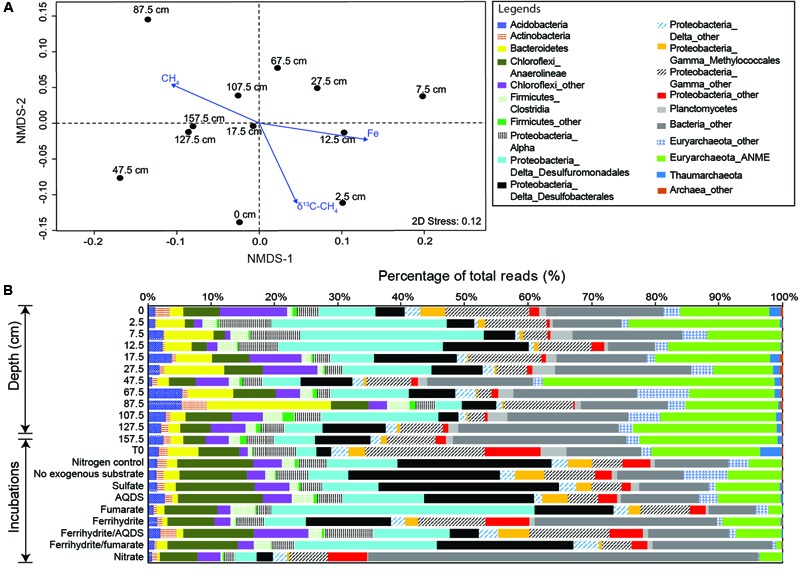
Community compositions and variations revealed by 16S rRNA gene amplicons. **(A)** Non-metric multidimensional scaling of community relatedness quantified by the weighted Unifrac matrix with the overlay of ordination for selected environmental parameters. **(B)** Abundances of major divisions for environmental and incubated samples.

The number of observed OTUs was the highest in the bubbling pool, decreased dramatically from the core top to the depth of 7.5 cm, and increased toward the bottom of the core (**Figure 1, Supplementary Figure S1** and Table S1). The trends of the Chao1 estimator and Inverse Simpson indices resembled the pattern of observed OTUs, with the highest values of both richness and evenness occurring in the bubbling pool (**Supplementary Figure S1**). Environmental vectors were fitted onto the ordination of NMDS (**Figure 2A**). The projections were significantly correlated with the concentrations of Fe (*R*^2^ = 0.60, *P <* 0.05), methane (*R*^2^ = 0.48, *P <* 0.05) or δ^13^C values of CH_4_ (*R*^2^ = 0.50, *P <* 0.05). Communities from 2.5, 7.5, and 12.5 cm (high dissolved Fe concentrations and δ^13^C values of CH_4_) were clearly separated from communities from 107.5, 127.5, and 157.5 cm (high concentrations of methane) along the first axis (**Figure 2A**).

### Community Compositions Based on 16S rRNA Gene Amplicons

A total of 18,401 bacterial and 3,510 archaeal OTUs (97% similarity), representing 157 classes (140 bacterial and 17 archaeal) within 44 phyla were recovered from all samples, with per sample OTU counts ranging from 1,622 to 6,207 (Supplementary Table S1). The dominant phyla include Proteobacteria (α, β, and γ divisions), Euryarchaeota, Chloroflexi, Firmicutes, Bacteroidetes, and Acidobacteria (∼79% of all sequences; **Figure 2B**). Planctomycetes, Actinobacteria, Lentisphaerae, OP8, Thaumarchaeota, JS1, Spirochaetes, and OD1 were present at a lower abundance (∼7% of all sequences).

The majority of bacterial reads were assigned to the orders Desulfuromonadales (14.8%) and Desulfobacterales (7.7%) within Deltaproteobacteria (Supplementary Table S6). Most sequences matching Desulfuromonadales were related to the families Sva1033, Desulfuromonadaceae, and Geobacteraceae. In particular, sequences affiliated with Desulfuromonadaceae constituted 26–29% of sequences in each sample at 2.5–12.5 cm depths, where methane concentrations were low and dissolved Fe and Mn concentrations were high. The dominant OTUs were classified to Sva1033 (Supplementary Table S7) and their abundances were highly correlated with the dissolved Fe and Mn concentrations (ρ = 0.78 and 0.75, respectively, *P* < 0.05).

In contrast, the majority of archaeal reads (18% of all reads) were assigned to the order Methanosarcinales within Methanomicrobia and primarily related to ANME-2 clades (9–36% in each sample) (**Figure 2B** and Supplementary Table S6). Most sequences (>98%) related to ANME-2 were phylogenetically assigned to the ANME-2a/2b group. The remaining 1% of ANME-2 related sequences shared 93% similarity with the nitrate-reducing anaerobic methane oxidizer, Candidatus *Methanoperedens nitroreducens* (Haroon et al., 2013). A few other sequences shared 99% similarity with the freshwater subgroup of ANME-1 from terrestrial subsurface environments in Japan (Takeuchi et al., 2011) and 97% similarity with ANME-1b from MVs in southwestern Taiwan (Cheng et al., 2012).

A proportion of archaeal sequences were related to Methanobacteriales, Methanococcales, Methanomicrobiales, Methanosarcinales, and Methanocellales. Their abundances were low at shallow depths (∼1% from the surface to the depth of 17.5 cm) and became abundant (5–8%) at depth intervals between 67.5 and 107.5 cm (**Supplementary Figure S2a** and Table S6). The dominant OTUs shared 99% and 97% similarity with *Methanocalculus pumilus* and *Methanosarcina semesiae*, respectively (Supplementary Table S7).

Sequences matching Alphaproteobacteria, Gammaproteo-bacteria, and Thaumarchaeota were abundant in samples with methane concentrations lower than 1 mM. Within Gammaproteobacteria, distinct families dominated at different depths. For example, *Methylomicrobium*-related sequences (within Methylococcales) were primarily distributed in the bubbling pool, whereas *Marinobacter*-related sequences (within Alteromonadales) were abundant in the core samples (Supplementary Tables S6, S7). For comparison, dominant phyla in samples with methane concentrations above 1 mM include Planctomycetes, Bacteroidetes, Actinobacteria, Acidobacteria, Chloroflexi (Anaerolineae), and Firmicutes (**Figure 2**). Among these phyla, sequences affiliated with *Prolixibacter denitrificans* MIC1-1 (96% identity) within Bacteroidetes constituted 18% of total reads and represent the dominant OTUs at 87.5 cm (Supplementary Table S7). Sequences of the other major OTUs were related to *Demequina* spp. ER-8 within Actinobacteria, *Thermotomaculum hydrothermale* strain AC55 within Acidobacteria, uncultured Ardenticatenia within Chloroflexi, and uncultured Clostridiales within Firmicutes (Supplementary Table S7).

### Taxonomic and Functional Variations Based on Metagenomic Data

**FIGURE 3:**
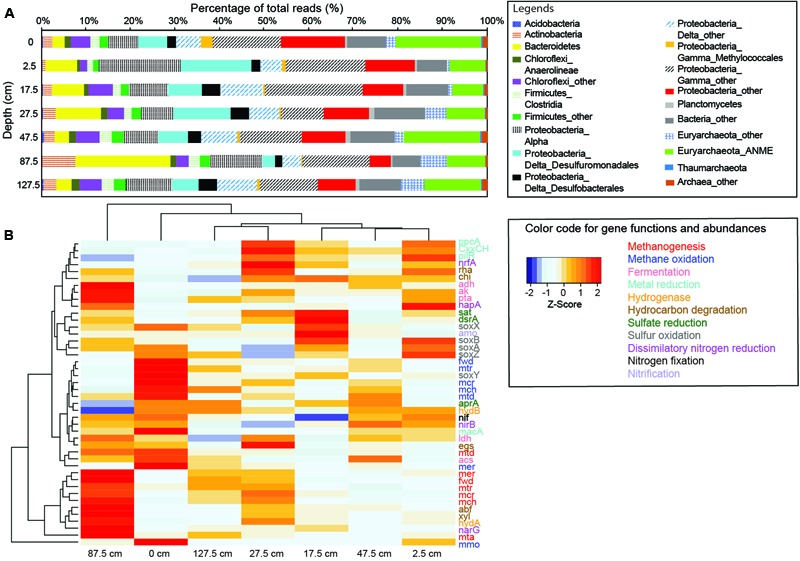
Community compositions and gene abundances revealed by metagenomic data. **(A)** Community compositions revealed by metagenomic reads classified against the NCBI-nr database. **(B)** Heat map of genes families associated with carbon, sulfur, metal, and nitrogen pathways.

A total of 0.8–1.7 million sequences per sample were obtained for further metagenomics analysis, and 21–32% of the sequences from each sample (373,020–769,841 reads per sample; Supplementary Table S1) were assigned to specific genes. A total of 28–34% of these annotated genes matched prokaryotic genes in the SEED (Supplementary Table S1). The majority of reads (>99%) were derived from prokaryotes (Supplementary Table S1). Therefore, protein coding sequences of prokaryotes were the main focus (**Figure 3A**). The odds ratios of specific single-copy genes in individual metagenomes to the average metagenome varied between 0.6 and 1.4 (**Supplementary Figure S3**). With the exceptions for some genes from 2.5 and 87.5 cm, most odds ratios were between 0.8 and 1.2. These results suggest that the sequencing bias is limited and the obtained metagenomes could be used to address the abundance variations. The abundances of key enzymes/functional genes were variable along depth (**Figure 3B**). Of all gene systems, AOM, fermentation and hydrolysis, and metal reduction were the most abundant (**Supplementary Figure S4**). The abundances of most investigated genes could not be differentiated statistically in accordance with the methane abundance category.

Abundances of the genes *mmo/pmo* in the bubbling pool and top sediments were above the average for all depths (**Figure 3B**). The majority of the genes *mmo/pmo* were affiliated with *Methylobacter* spp. (sharing 80–96% identity) and *Methylohalobius crimeensis* (sharing 80–98% identity) within Methylococcaceae (Supplementary Table S8). The abundances of the genes *mcr, mtr, fwd, mch, mer*, and *mtd* affiliated with ANME members were highest in the bubbling pool and above the average at 27.5 and 47.5 cm (**Figure 3B**). These genes shared 80–95% similarity with the ANME-2a collected from Capt Aryutinov MV (Wang F.-P. et al., 2014), and constituted the majority of archaeal reads (∼10% of all reads). In contrast, the same set of genes affiliated with methanogens (mostly Methanosarcinales) was more abundant at 87.5 and 127.5 cm than the average for all depths (**Figure 3B**). Sequences representing two MHC genes with predicted S-layer domains were detected in seven samples and shared 60–80% similarity with ANME-2a. However, the relative abundances of the two genes were not particularly high (0.03–0.05%).

Genes *ak* and *pta* (for synthesis of acetate from acetyl-CoA), genes *adh* and *ldh* (for production of alcohol and lactate), and gene *hyd*A (for H_2_ production) were more abundant at 87.5 cm than the average for all depths (**Figure 3B**). In contrast, gene *acs* (for acetate synthesis from acetyl-CoA) and gene *hyd*B (for the oxidation of H_2_) were more abundant in the bubbling pool than the average for all depths. Most of these reads were affiliated with *Clostridium* (for gene *ak*), *Desulfuromonas* (for gene *pta*), *Bacillus* (for gene *adh*), *Gluconobacter* (for gene *ldh*), Candidatus *M. nitroreducens* (for gene *acs*), *Halanaerobium saccharolyticum* (for gene *hydA*), and *Geobacter* spp. (for gene *hydB*) (Supplementary Table S8).

MacA represents an intermediate carrier between electron transfer components in the inner and outer membranes (Butler et al., 2004). Its abundances in the bubbling pool and at 2.5, 47.5, and 127.5 cm were above the average for all depths (**Figure 3B**). In contrast, the abundances of genes *pil*R, which encodes an enhancer binding protein involved in Fe(III) respiratory functions, and *ppc*A, an intermediary carrier for electron transport from acetate to terminal Fe(III) reductases in the outer membrane (Lloyd et al., 2006; Juárez et al., 2009), and genes containing CxxCH heme-binding motif at 2.5, 17.5, and 27.5 cm were above the average for all depths (**Figure 3B**). Most reads of *pil*R, *ppc*A, and CxxCH were affiliated with *Geobacter* (for *mac*A and *pil*R), *Geoalkalibacter* (for *mac*A), *Desulfuromonas* (for *CxxH*) and *Pelobacter* (for *mac*A) within Desulfuromonadales (Supplementary Table S8).

Dissimilatory sulfate reduction was inferred from the genes *apr*A [for conversions of adenosine-5’-phosphosulfate (APS) to AMP and sulfite], *sat* (for conversions of activated sulfate to APS), and *dsr*AB (for reduction of sulfite to sulfide) (**Figure 3B**). The abundances of *apr*A, *dsr*AB, and *sat* at 17.5 cm were above the average for all depths (**Figure 3B**). Less than 3% of sequences encoding *apr*A were related to sulfur-oxidizing bacteria and 37% to AprA lineages I, 20% to Desulfobacteraceae, and 40% to Desulfobulbaceae. Reads of *dsr*AB were primarily related to Desulfobulbaceae and Desulfobacteraceae within Desulfobacterales. Additionally, 25% of *dsr*AB reads were affiliated with Peptococcaceae within Clostridiales (Supplementary Table S8). Reads of reverse *dsr*AB involved in aerobic and photosynthetic sulfur oxidation (Eisen et al., 2002; Ghosh and Dam, 2009; Loy et al., 2009) were affiliated with *Thiothrix* and *Halorhodospira*, respectively, and accounted for ∼4% of all *dsr*AB reads. The abundances of sulfur oxidation gene system (*sox*ABYX) in the bubbling pool and at 2.5 and 17.5 cm were above average for all depths (**Figure 3B**). These reads shared 60–80% identity with *Thiolapillus brandeum* (Supplementary Table S8) isolated from the Okinawa Trough (Nunoura et al., 2014).

For dissimilatory nitrate reduction, the abundances of gene *nar*G (which encodes the membrane-bound nitrate reductase) at 17.5 and 87.5 cm were above the average for all depths, whereas those of gene *nap*A (which encodes the periplasmic nitrate reductase) at 2.5 and 17.5 cm were above the average for all depths. Both types of nitrite reductase (*nir*B and *nrf*A) catalyzing the reduction of nitrite to ammonium were more abundant at 2.5 and 17.5 cm than the average for all depths. Although diverse *nar*G genes were detected, the majority of *nar*G reads were affiliated with *Marinobacter* spp. (Supplementary Table S8). The abundances of *nif* genes for di-nitrogen fixation in the bubbling pool and at 2.5 cm were above the average for all depths (**Figure 3B**). Reads of *nif* genes (*nif*D, *nif*H, and *nif*K) were related with a variety of microorganisms and the majority were affiliated with *Methanosarcina* and *Methanolobus* (Supplementary Table S8).

### Incubations for AOM Activity

**FIGURE 4:**
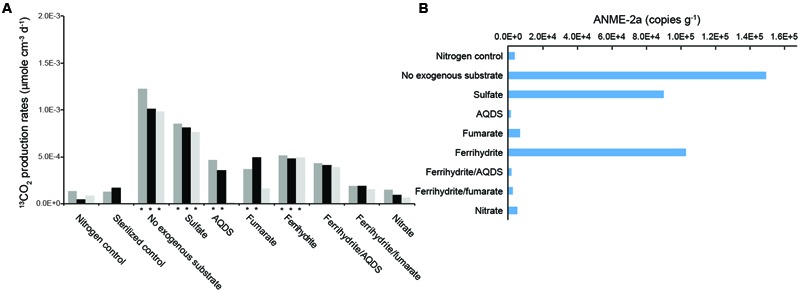
AOM rates **(A)** and abundances of ANME-2a **(B)** under different treatments. Time course measurements and regression analyses are provided in **Supplementary Figure S4**. ^∗^ symbol denotes the AOM rates significantly different from the sterilized control (*P* < 0.05).

Most incubations yielded a steady increase of the accumulated ^13^CO_2_ with time (*R*^2^ > 0.5; **Supplementary Figure S5**). In contrast, the nitrogen control and incubations with ferrihydrite/AQDS, ferrihydrite/fumarate, and nitrate were characterized by different degrees of fluctuations in ^13^CO_2_ amount over time. Therefore, the rates derived from the regression analyses were calculated for individual incubations. High AOM activities at rates between 9.8 × 10^-4^ and 1.2 × 10^-3^ μmol cm^-3^ d^-1^ and between 7.6 × 10^-4^ and 8.5 × 10^-4^ μmol cm^-3^ d^-1^ were obtained for the incubations without exogenous electron acceptors and the ones supplied with sulfate, respectively (**Figure 4A** and **Supplementary Figure S5**). In the presence of ferrihydrite, fumarate and AQDS, the rates ranged from 4.8 × 10^-4^ to 5.2 × 10^-4^, from 1.6 × 10^-4^ to 4.9 × 10^-4^, and from 1.9 × 10^-5^ to 4.7 × 10^-4^ μmol cm^-3^ d^-1^, respectively. With the exceptions for one individual incubation with fumarate and AQDS, these rates were statistically different from the average rate of the sterilized control (1.5 × 10^-4^ μmole cm^-3^ d^-1^) (*P <* 0.05). In contrast, in the presence of ferrihydrite/AQDS, ferrihydrite/fumarate, and nitrate, the ^13^CO_2_ was produced at a rate statistically no different from the sterilized control, and therefore considered negligible. The copy numbers of ANME-2a increased in the incubations with no exogenous electron acceptor (1.50 × 10^5^ copies g^-1^ of slurries), sulfate (9.04 × 10^4^ copies g^-1^ of slurries) and ferrihydrite (1.03 × 10^5^ copies g^-1^ of slurries) when compared with the nitrogen control (**Figure 4B**). The copy numbers of ANME-2a in other incubations were either marginally higher or less than that in the nitrogen control.

The two-dimensional PCoA plot revealed that communities clustered primarily by the addition of electron acceptors (**Supplementary Figure S2b**). The community at the start of incubation was more similar to the communities in the bubbling pool and sediment core, and the addition of electron acceptors and methane shifted community compositions by various degrees after incubation for 176 days (**Figure 2B** and **Supplementary Figure S2b**). Desulfobacterales members appear to be the major group for the incubations without exogenous electron acceptors, or with sulfate or AQDS. The dominant OTU in these incubations was classified into the genus *Desulfurivibrio* (Supplementary Table S9) and shared 90% similarity with *Desulfurivibrio alkaliphilus* (AHT2) from a soda lake in Egypt (NR_074971.1). The incubations supplied with fumarate or ferrihydrite/fumarate were characterized by abundant reads sharing 98% similarity with *Pelobacter massiliensis* (HHQ7) (Supplementary Table S9), a strict anaerobe capable of fermenting l,2,4-trihydroxybenzene to acetate and reducing oxidized metal (Schnell et al., 1991). The dominant OTU in the incubations supplied with ferrihydrite or nitrate shared 93% similarity with the uncultured clone (AB247832) collected from hydrothermal vents in Lau Basin.

ANME-2a/2b reads dominated the archaeal communities in all incubations (**Figure 2B** and **Supplementary Figure S2a**). Reads affiliated with *Methanococcus* spp. and *Methanosaeta* spp. were abundant in the incubations without exogenous electron acceptors and those supplied with fumarate, respectively. For the other incubations, the proportion of methanogens was lower than 1% of total reads (**Supplementary Figure S3**).

## Discussion

### Geochemical Stratification

The methane, DIC, and isotopic profiles are consistent with the compartmentalization of methane metabolisms at discrete depth intervals. At depths shallower than 20 cm, low methane concentrations, greater δ^13^C values of CH_4_, high DIC concentrations, and low δ^13^C values of DIC indicate the presence of methane oxidation. At the deeper intervals, the contrasting geochemical characteristics point to the predominance of methanogenesis over methane oxidation. Furthermore, dissolved Mn and Fe are generally dominated by their reduced forms [Mn(II) and Fe(II)] under near-neutral pH. Their high concentrations in the top portion of the core suggests that the pore water has to be anoxic. Methane oxidation prevailing at these intervals would be anaerobic. Resembling the findings of previous studies (Chang et al., 2012; Wang P.-L. et al., 2014), methane concentrations were inversely correlated with Fe/Mn concentrations (ρ = –0.65 and -0.65, respectively, *P* < 0.05). In contrast, the δ^13^C values of CH_4_ and the concentrations of dissolved Mn and Fe showed a strong covariance (ρ = 0.71 and 0.69, respectively, *P* < 0.05). These geochemical characteristics mark the transition of Fe/Mn to methane at depths of less than 20 cm, suggesting that AOM might be linked directly or indirectly to microbial manganese/iron reduction through a pathway that remains uncharacterized thoroughly (Chang et al., 2012; Wang P.-L. et al., 2014).

### Methane Oxidation and Metal Reduction

Interpretation with regard to AOM and related metabolisms based on geochemical evidence is comparable with that based on molecular evidence obtained from this and previous studies (Chang et al., 2012; Wang P.-L. et al., 2014). The copy numbers of ANME-2a 16S rRNA genes were enhanced greatly at the methane transition intervals (<20 cm) (**Figure 1**). Furthermore, genes responsible for the AOM processes, including *mcr, mtr, fwd, mch, mer*, and *mtd*, were all recovered from metagenomic data and phylogenetically classified into the ANME-2a lineage (**Supplementary Figure S6**). These lines of evidence combined with positive AOM activities derived from incubation experiments (**Figure 4** and **Supplementary Figure S5**) and geochemical characteristics (**Figure 1**) suggest that the anoxic sediments near the surface harbored substantial amounts of anaerobic methane oxidizers that actively oxidized methane produced at the adjacent depth intervals.

Of all the incubation sets, the rate of AOM without exogenous electron acceptors appeared to be the fastest (**Figure 4A**). The results suggest that AOM could proceed with the reduction of electron acceptors within sediments. Considering that sulfate and nitrate in pore water and the bubbling pool were below the detection, their abundances in the incubated slurries would be further diluted by the basal solution added. Therefore, the most plausible electron acceptor, if any, would be the oxidized iron associated with sediments. This inference is, however, not supported by the lower AOM rates obtained from the incubations with synthetic ferrihydrite when compared with the rates for treatments without an exogenous electron acceptor. Previous studies indicate that ferrihydrite is the least crystalline form of iron oxide commonly observed in natural samples (Waite et al., 1994) and, therefore, has the greatest bioreactivity for microbial iron reduction. If ferric iron is the target electron acceptor, AOM would have to use the solid form distinct from the ferrihydrite supplemented with incubations. Mineralogical investigations are warranted to attest this assertion.

The AOM activities detected for incubations (excluding ferrihydrite/AQDS, ferrihydrite/fumarate, and nitrate) also suggest that a wide range of electron acceptors could at least maintain the viability of ANME-2a members to various degrees (**Figure 4**). The results are partially comparable with the previous study in which sulfate, AQDS, ferric citrate, ferrihydrite, humic acids, and melanin could feasibly stimulate AOM activity in sediments retrieved from a marine seep and a canal (Ettwig et al., 2016; Scheller et al., 2016). Our results additionally suggest that no exogenous addition, and additions of fumarate were also candidate treatments to facilitate the AOM. Furthermore, the 16S rRNA gene analysis conducted for incubated slurries yielded a predominance of ANME-2a members for positive AOM incubations (**Supplementary Figure S3**). The ANME-2a members have been experimentally demonstrated to exploit AQDS alone or to form syntrophic partnerships with Deltaproteobacteria for sulfate reduction (Scheller et al., 2016). The capability of ANME-2a members in exploiting all the other electron acceptors tested in this study has not been confirmed in previous studies.

Geochemical characteristics indicated that iron and manganese reduction prevailed in the top 40 cm of sediments (**Figure 1**). Such inferences were further supported by the results that Desulfuromonadales within Deltaproteobacteria constituted the most abundant order based on the 16S rRNA gene amplicon analyses. Its abundances were positively correlated with the concentrations of dissolved Mn and Fe. Strains belonging to Desulfuromonadales are known to be capable of catalyzing anaerobic respiration with nitrate, elemental sulfur, ferrihydrite, or manganese oxide as electron acceptors (Köpke et al., 2005; Greene et al., 2009; Summers et al., 2010; Greene, 2014). Members within Desulfuromonadales, such as *Geobacter*, rely on the outer membrane *c-*type cytochromes to transfer electrons to Fe(III) and Mn(IV) oxides (Lovley, 1993; Mehta et al., 2005). The great abundances of *c-*type cytochrome encoded genes (*mac*A, *ppc*A, and *CxxCH*) phylogenetically affiliated with *Geobacter* and *Pelobacter* further revealed the prevalence of metal reduction at the top 40 cm (**Figure 3** and Supplementary Table S8).

The coincidence of AOM and the metal reduction zone at similar depth intervals has been observed in this and previous studies for cores collected on different dates (2009 vs. 2010). These results suggest the persistence of a possible linkage between AOM and metal reduction through time. AOM driven by iron reduction has only been experimentally demonstrated in enrichment cultures composed of 40–50% “Candidatus *M. nitroreducens*” of the ANME-2d group and 40% *Methylomirabilis oxyfera* of NC-10 phylum (Ettwig et al., 2016). Additional incubations with *M. oxyfera* as the sole methane oxidizer did not yield the production of detectable ferrous iron, leading Ettwig et al. (2016) to conclude that AOM coupled with the production of ferrous iron was mediated solely by *M. nitroreducens*. However, the 16S rRNA genes related to *M. nitroreducens* constituted only a minor portion (<1%) of the total reads in any environmental or incubated samples described in this study (**Supplementary Figure S3**), thereby precluding the attribution of the observed coincidence of AOM and metal reduction zone to the ANME-2d members.

The enhanced abundances of ANME-2a and Desulfuromonadales in the metal-to-methane transition zone provides the possibility that these two populations might form syntrophic consortia to facilitate the electron transport during AOM. To date, DIET has been proposed as the principal mechanism to couple the electron transport from AOM to sulfate reduction (McGlynn et al., 2015; Wegener et al., 2015). Through mining of ANME genomes and metagenomes, McGlynn et al. (2015) found that the genomes of ANME-2a/2b/2d groups encode large MHCs (up to 34 hemes). In particular, the MHCs of ANME-2 members were fused with the single putative S-layer domain. Such genetic organization together with cytochrome reactive staining and transmission electron microscopic observation led McGlynn et al. (2015) to propose that tandem proteins encoding formate dehydrogenase-related cytochrome *b* with MHCs and MHC/S-layer fusion proteins could be an effective route to facilitate interspecies transport of electrons generated from the oxidation of methane to the downstream product, reduced methanophenazine, thereby stabilizing syntrophic aggregate arrangements. It is likely that the MHC/S-layer fusion proteins might be also able to facilitate the reduction of metal oxides by ANME members without the need of metal reducers. Similarly, evidence from cultivation, expressions of genes (c-type cytochromes, *mcr*A, *dsr*A, and *pil*A), and visualization of microstructures has shown that the HotSeep-1 members could have produced nanowires, such as pili, extending to the surface of ANME-1 cells under the AOM conditions (Wegener et al., 2015). Again, such structures provide a putative route that enables the DIET between anaerobic methane oxidizers and metal reducers, and might facilitate the formation of syntrophic consortia with loose contact between individual cells (Wegener et al., 2015).

The MHCs/S-layer fusion proteins of ANME-2a (McGlynn et al., 2015) were present in all metagenomes of LGHMVs. If the MHCs/S-layer fusion protein indeed accounts for the electron transfer for the detected ANME-2a, syntrophic consortia could form with the aid of possible interspecies electron transport through the mechanisms describe above. Alternatively, electrons generated from anaerobic methane oxidizers could be transferred directly to extracellular electron acceptors in a process similar to that conducted by *G. sulfurreducens* (Methé et al., 2003) and possibly by ANME-2d (Ettwig et al., 2016). The exact interactions between the ANME and Desulfuromonadales members remain uncertain, as attempts to perform fluorescence *in situ* hybridization experiments were not successfully, likely due to the interference of sediment auto-fluorescence and low RNA content (unpublished results).

Finally, 16S rRNA genes related to *Methylomicrobium* of Methylococcaceae appear to be abundant in the bubbling fluids (1.4%, compared to the lowest value of 0.04% at 47.5 cm). Similarly, the abundances of *mmo* were more abundant in the bubbling fluids than that in cored sediments. Most genes of *mmo* were affiliated with *M. crimeensis* within Methylococcaceae. Strains related to these genera are capable of oxidizing methane under oxic conditions. Since the bubbling fluids were generally thought to originate from mineral dehydration or sediment compaction at great depths, the fluids are regarded the least altered and representative of the reducing source condition. The presence of these aerobic methane oxidizers, however, suggests that atmospheric oxygen might be entrained into the pool fluids through gas bubbling at quantities sufficient to drive methane oxidation while outcompeting the kinetics of abiotic oxidation of other reducing compounds (e.g., ferrous iron). The oxygenation induced by gas bubbling has been observed in intertidal sediments near the seep (O’Hara et al., 1995). Such a process would lead to a steeper redox gradient that facilitates the coexistence of metabolisms with a spectrum of oxygen affinities. Nevertheless, while methane emanating from the pool could be directly discharged into the atmosphere, the observed aerobic methane oxidizers could act as biological filters for methane discharge. For comparison, control of methane emitted from the mud platform adjacent to the pool would be primarily accounted for by the anaerobic methane oxidizers (e.g., ANME-2a members).

### Hydrolysis, Fermentation, and Sulfur Cycling

A portion of DOC is formed through the hydrolysis and fermentation of organic matter catalyzed by microorganisms and fuels electron-accepting processes downstream the metabolic network. Therefore, DOC concentrations represent the interplay between the production and consumption processes mediated by different components of microbial populations. The low DOC concentration at shallow depth suggests that the production of DOC could have been efficiently channeled to metal reduction and perhaps AOM. In contrast, the high DOC concentration at deeper intervals suggests that the DOC cycling might not be as efficient as at shallow depths due to the production of different intermediate DOC compounds and/or the shift in microbial communities and functions. The enhanced proportions of Bacteroidetes, Firmicutes, Chloroflexi, and Actinobacteria, and the increased DOC concentrations at depth provide a link between the DOC accumulation and these taxonomic groups. The 16S rRNA gene sequences of the dominant OTUs were affiliated with *P. denitrificans, S. paucivorans*, uncultured Ardenticatenia, and *Demequina* sp. ER-8. These closest strains are generally considered to be capable of degrading organic matter.

For the degradation of specific categories of organic matter, the 16S rRNA gene sequences related to *Mycobacterium* within Actinobacteria and *Fulvivirga* within Bacteroidetes were inferred to represent community members capable of degrading chitin. Sequences 16S rRNA genes related to *Prolixibacter* within Bacteroidetes were inferred to represent community members capable of degrading plant-derived materials. Similarly, the greater abundances of genes *rha, abf*, and *xyl* compared to *chi* at depths between 77.5 and 97.5 cm were correlated with the increased concentrations of DOC, suggesting an enhanced capability for the decomposition of plant-derived materials primarily mediated by Bacteroidetes. Finally, the proliferation of fermenters, such as *Clostridium, Bacillus*, and *Lactobacillus*, which are known of being capable of synthesizing hydrocarbons from acetate, butyrate, and alcohol, might also contribute to the increased concentrations of DOC.

Based on the classification of 16S rRNA amplicons (**Supplementary Figures S2a**), the dominant methanogens at 67.5 cm were related to *M. pumilus*, a strain known for using H_2_/CO_2_ for methane production (Mori et al., 2000). In contrast, the abundances of acetoclastic *Methanosaeta harundinacea* (Rotaru et al., 2013) increased to be almost equal to those of *M. pumilus* at 87.5 cm. The variable proportions of methanogen-related sequences along depth combined with methane isotopic compositions and DOC profiles suggest that that predominant methanogenic pathways (either CO_2_-reducing or acetoclastic) shifted at different depths, a metabolic scheme potentially driven by the end products from organic hydrolysis, degradation, and fermentation.

The metagenomic results also indicated that reads classified to *sox*ABYX system, *apr*A, and *dsr*AB were more abundant at 17.5 cm when compared with those at other depths. This combined with the detection of a small proportion of *apr*A genes taxonomically assigned to AprA lineage I composed of sulfur oxidizers, and *Desulfocapsa sulfexigens* capable of disproportionating inorganic sulfur compounds (Finster et al., 2013), and abundant *dsrAB* genes assigned to Desulfobacterales and Desulfobulbaceae suggest that oxidative and reductive pathways of sulfur coexist at the depth interval geochemically interpreted as the anoxic zone. The coexistence of both sulfur oxidizers and sulfate reducers has been observed at depths below sulfate reduction zone, and inferred to catalyze the “cryptic” sulfur cycling based on the gene expression, rate measurement, and isotopic pattern (Wasmund et al., 2017). It remains unclear whether such sulfur cycling also prevails at our investigated site.

## Conclusion

Patterns of geochemical characteristics, gene abundances and assemblages, and incubation responses suggest a wide range of metabolic capability and interactions for microbial communities thriving at the LGHMV. The metabolic interactions are primarily driven by the upstream degradation of complex organic matter (e.g., chitin and cellulose) and fermentation mediated by Bacteroidetes, Chloroflexi, and Firmicutes at depth. Despite the fact that a portion of degradative and fermentative products in mobile phase are accumulated in pore water, some are further exploited by various methanogens, fueling downstream AOM processes catalyzed by the ANME-2a members near the surface. While the AOM could be stimulated by a range of electron acceptors, their activity is intimately linked with iron reduction likely through intercellular electron transfer under the *in situ* conditions. The cascading transformation of organic carbon highlights the importance of the microbial metabolic network in cycling sediment-associated, recalcitrant organic matter and controlling methane emission in terrestrial ferruginous, sulfate-depleted MVs.

## Author Contributions

T-HT, L-HL, and P-LW designed research, and analyzed and interpreted the data. L-WW was involved in field sampling and performed part of molecular analysis. Y-SL performed part of aqueous chemistry. HI performed part of molecular analysis. All authors participated in writing the manuscript.

## Conflict of Interest Statement

The authors declare that the research was conducted in the absence of any commercial or financial relationships that could be construed as a potential conflict of interest.

## Footnotes

1 https://www.arb-silva.de

## Supplementary Material

FIGURE S1 Chao1 and Inverse Simpson estimators as a function of depth. Data points are mean values based on rarefaction at a standardized sequence count (*n* = 10,008) with bars indicating 95% confidence intervals.

FIGURE S2 **(a)** Proportions of 16S rRNA gene amplicons related to methanogens and ANME members in environmental and incubated samples. **(b)** PCoA analysis for community variance among environmental and incubated samples.

FIGURE S3 Odds ratios for the 37 single-copy housing-keeping genes between individual metagenomes and the average metagenome.

FIGURE S4 Relative abundances of gene families listed in **Figure 3B**. Abundances were calculated as read count per gene per base of gene length, and shown as a proportion against the abundance of the universal single-copy gene encoding RNA polymerase subunit B (*rpo*B).

FIGURE S5 Accumulations of ^13^CO_2_ over time for incubations with different treatments. The dotted lines represent the regression of data points with *R*^2^ values shown in color corresponding to individual replicates or control.

FIGURE S6 Phylogenetic tree of *mcr*A genes. Sequences retrieved from the metagenomes with a length of >300 bp are labeled in blue, whereas sequences previously reported for other cores in LGHMVs are labeled in green. The accession numbers of reference sequences are in brackets.
