# Supplementary figures and images for: Microbial Community Composition and Functional Capacity in a Terrestrial Ferruginous, Sulfate-Depleted Mud Volcano
Source: Front Microbiol. 2017 Nov 2;8:2137. doi: 10.3389/fmicb.2017.02137 (PMC5673622; doi:10.3389/fmicb.2017.02137)

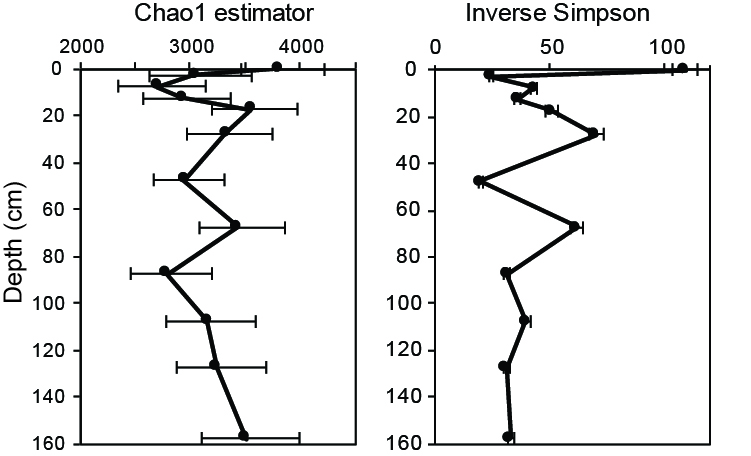

Supplement: FIGURE S1 — Chao1 and Inverse Simpson estimators as a function of depth. Data points are mean values based on rarefaction at a standardized sequence count (n = 10,008) with bars indicating 95% confidence intervals. [file Image_1.JPEG]

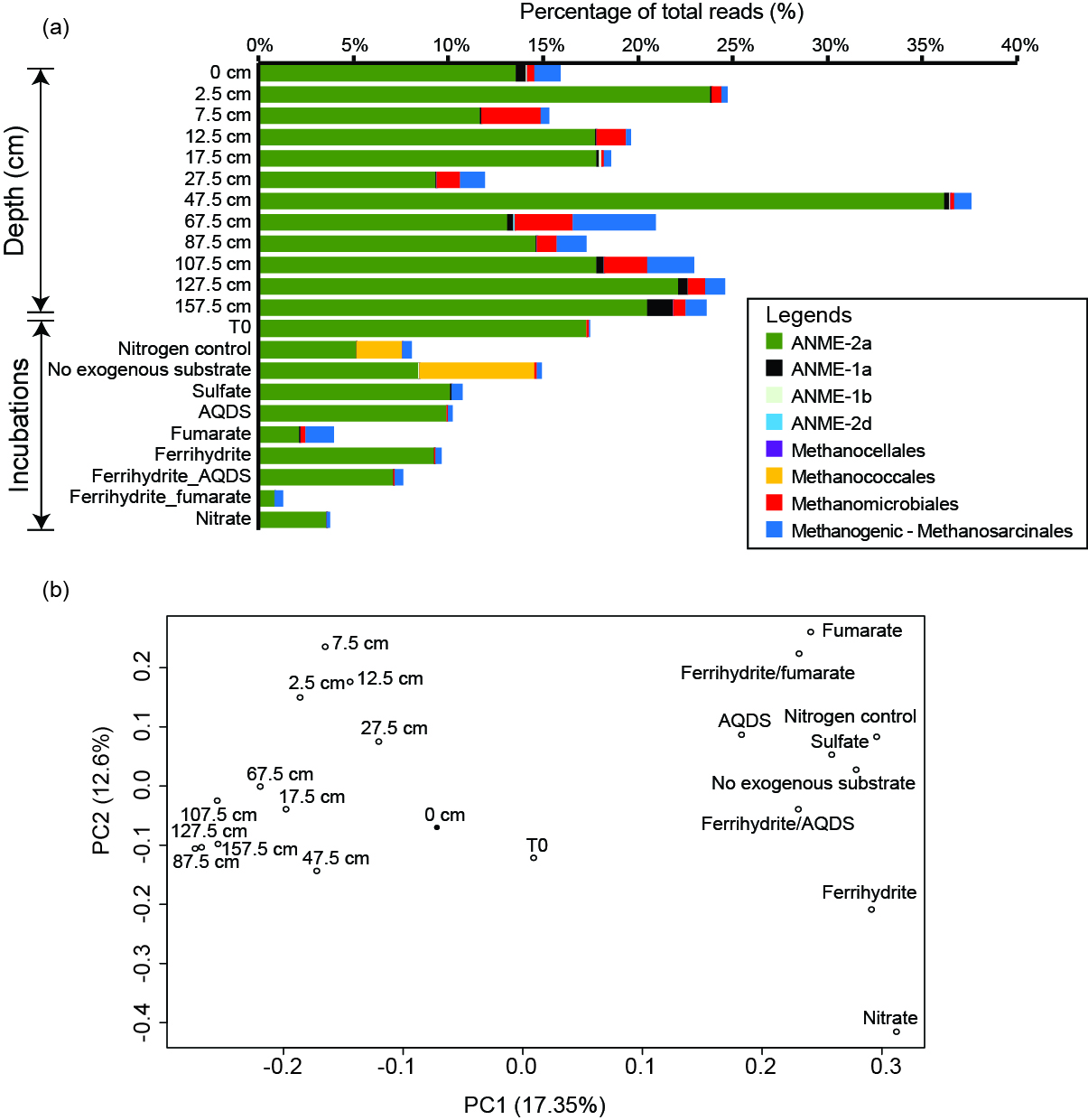

Supplement: FIGURE S2 — (a) Proportions of 16S rRNA gene amplicons related to methanogens and ANME members in environmental and incubated samples. (b) PCoA analysis for community variance among environmental and incubated samples. [file Image_2.JPEG]

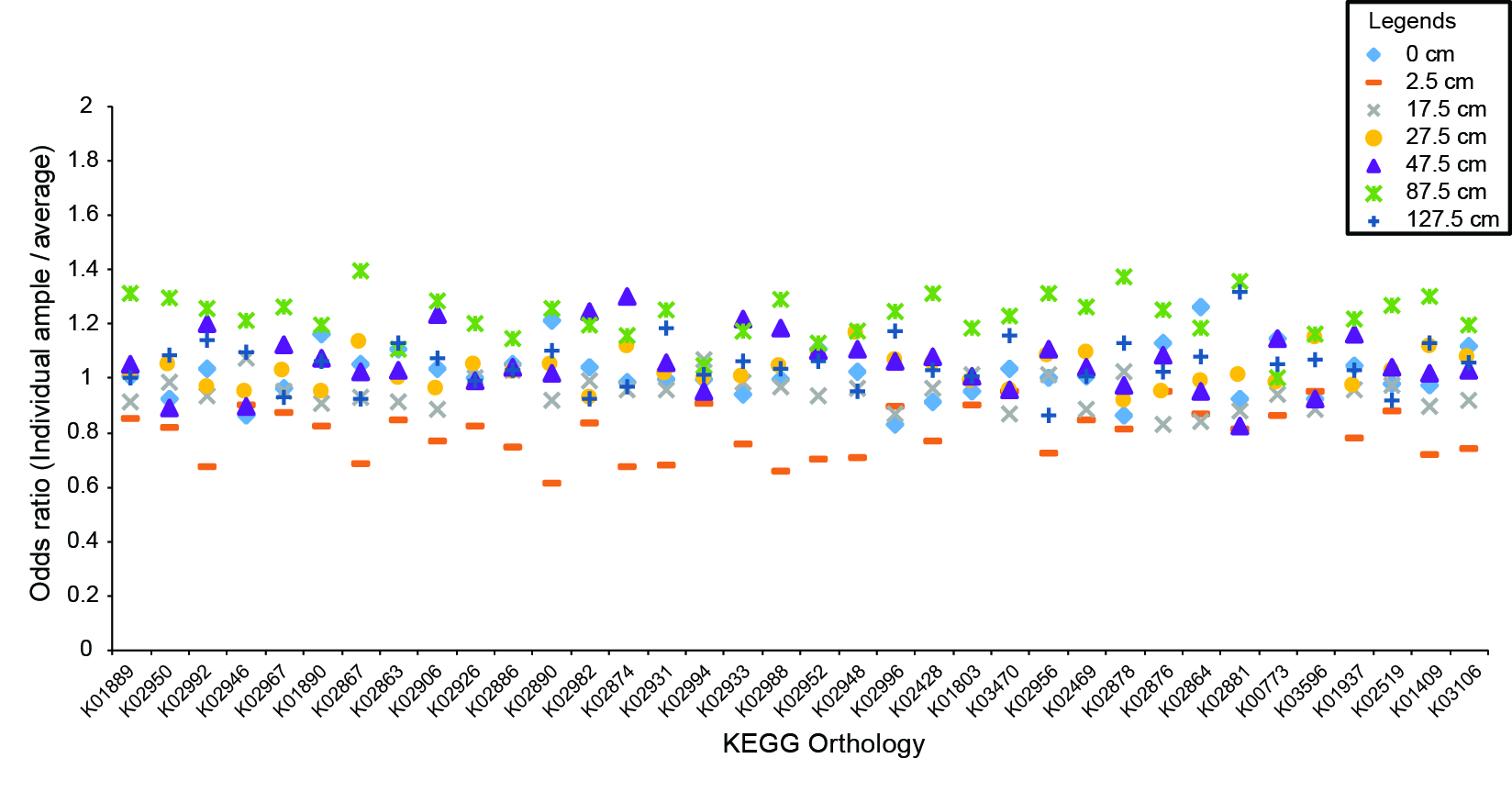

Supplement: FIGURE S3 — Odds ratios for the 37 single-copy housing-keeping genes between individual metagenomes and the average metagenome. [file Image_3.JPEG]

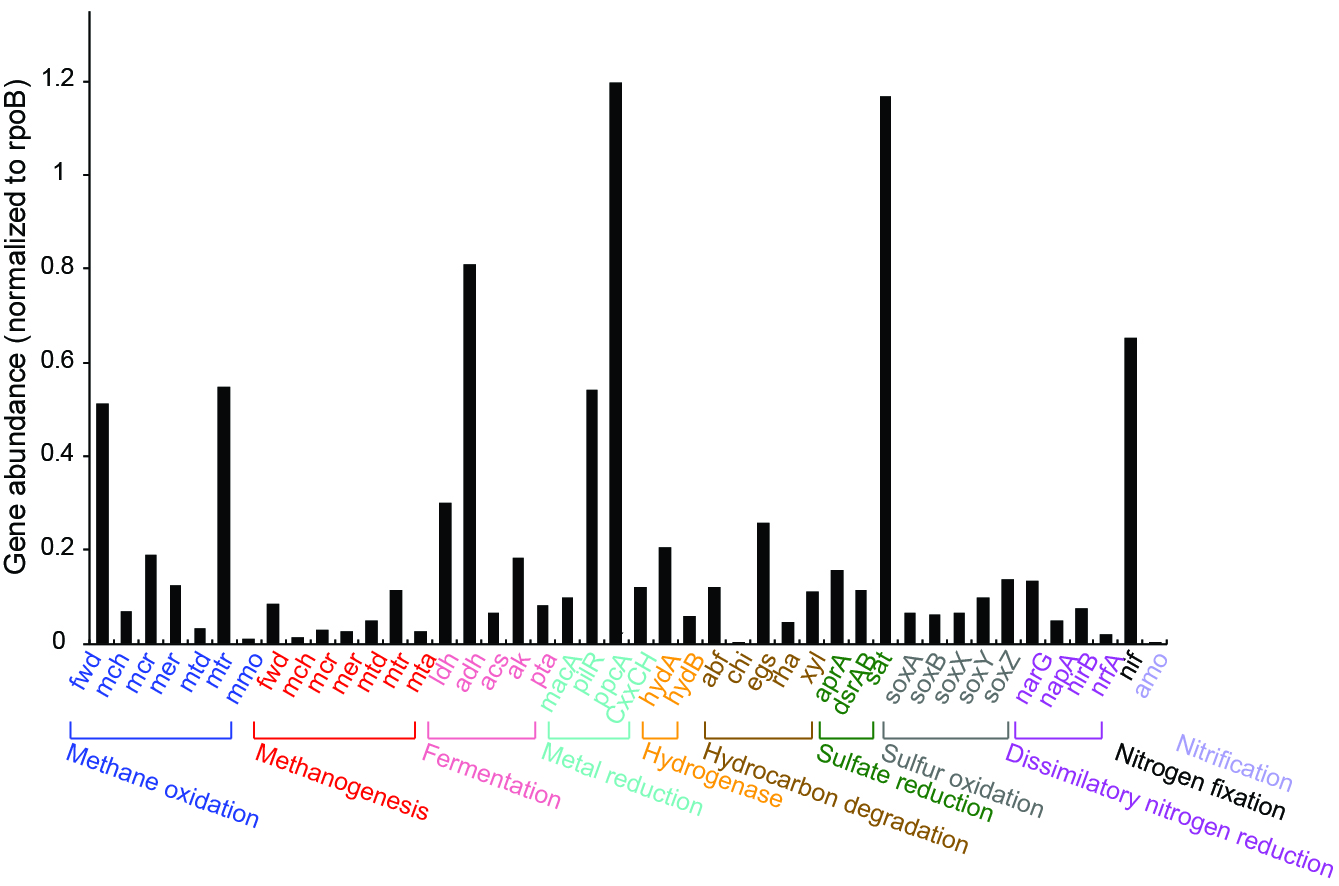

Supplement: FIGURE S4 — Relative abundances of gene families listed in Figure 3B. Abundances were calculated as read count per gene per base of gene length, and shown as a proportion against the abundance of the universal single-copy gene encoding RNA polymerase subunit B (rpoB). [file Image_4.JPEG]

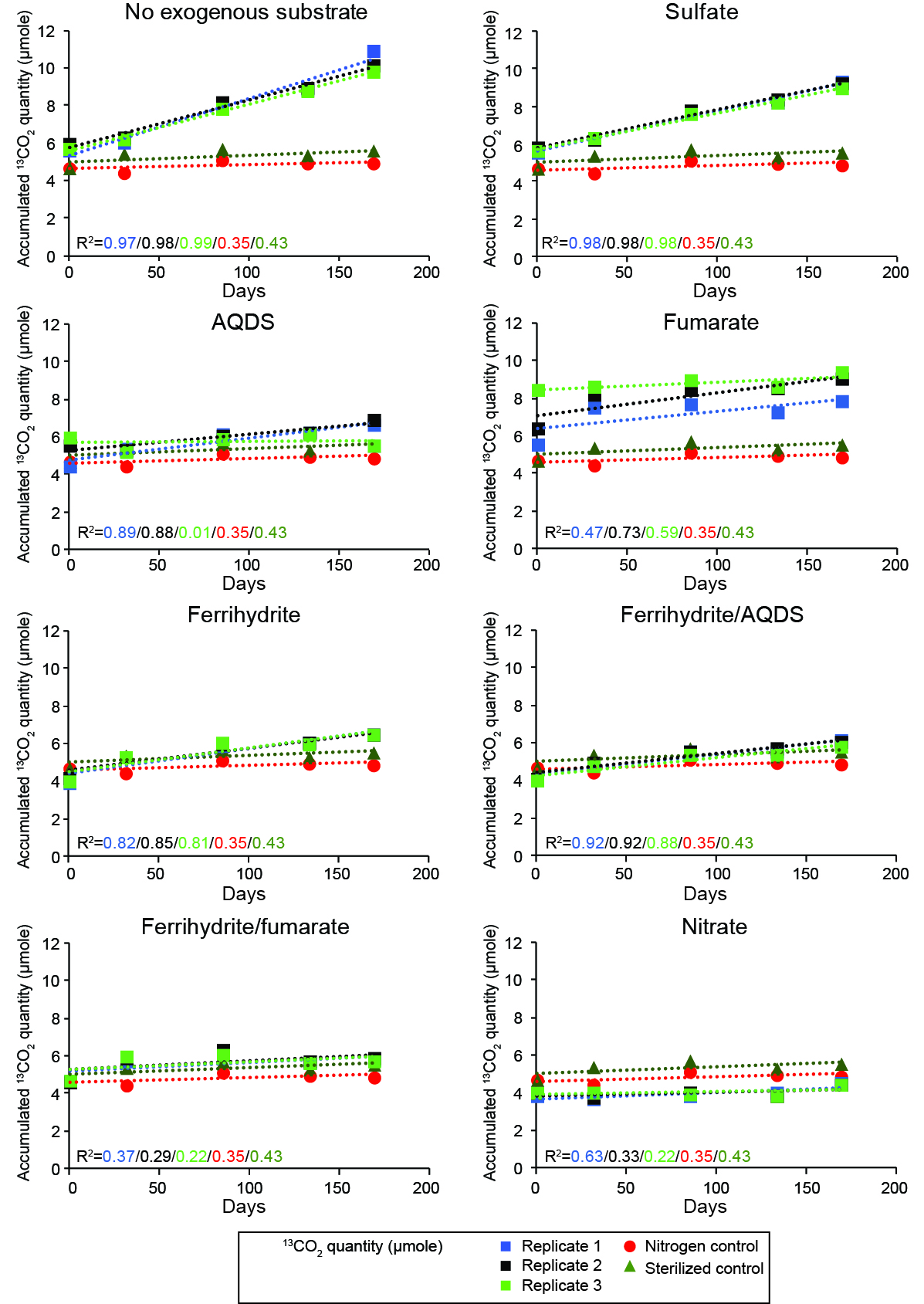

Supplement: FIGURE S5 — Accumulations of 13CO2 over time for incubations with different treatments. The dotted lines represent the regression of data points with R2 values shown in color corresponding to individual replicates or control. [file Image_5.JPEG]

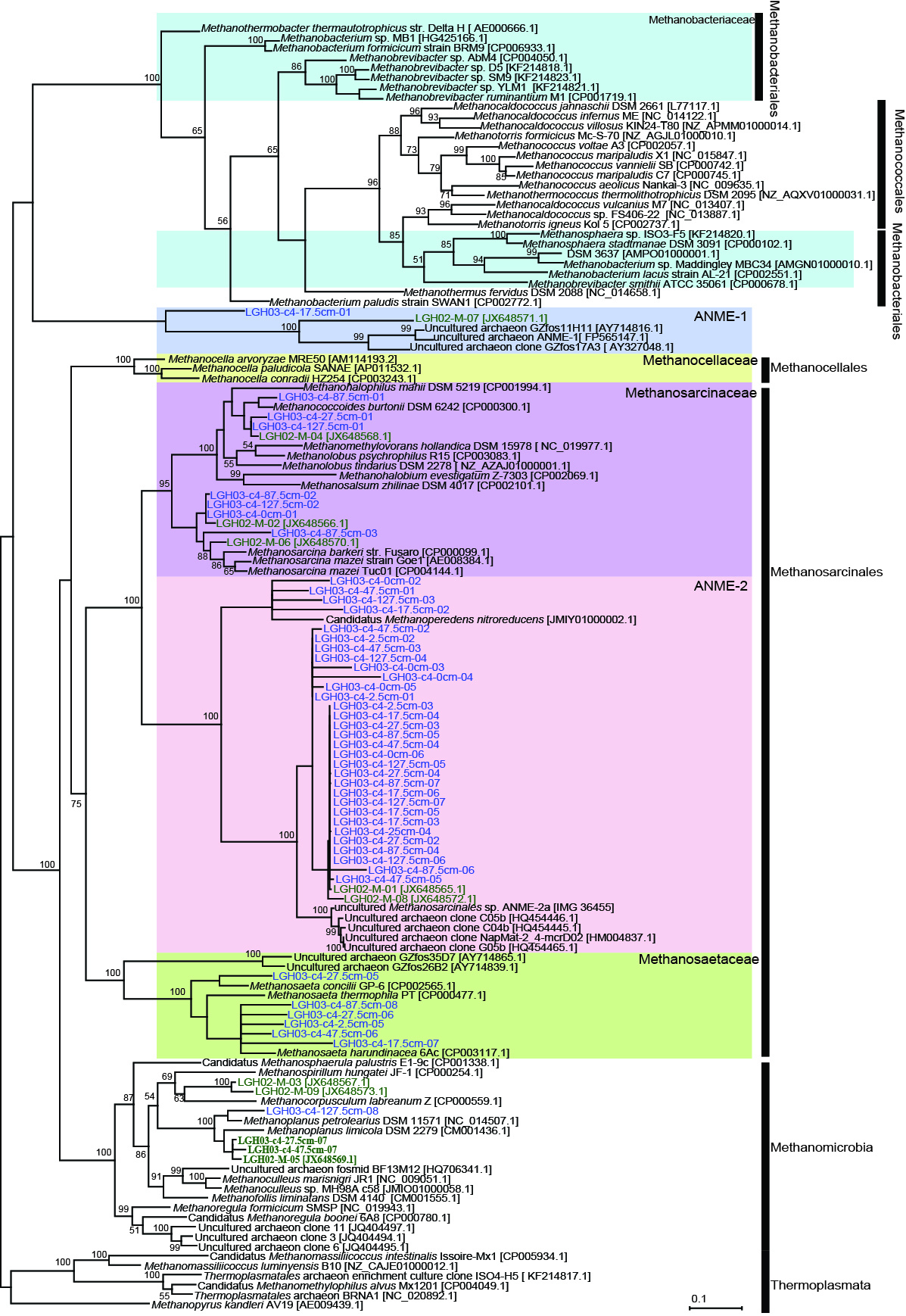

Supplement: FIGURE S6 — Phylogenetic tree of mcrA genes. Sequences retrieved from the metagenomes with a length of >300 bp are labeled in blue, whereas sequences previously reported for other cores in LGHMVs are labeled in green. The accession numbers of reference sequences are in brackets. [file Image_6.JPEG]
